# High efficacy of Azacitidine plus HAG in acute myeloid leukemia: an open-label, single-arm, multi-center, phase 2 study

**DOI:** 10.1038/s41408-022-00740-3

**Published:** 2022-10-28

**Authors:** Jun Li, Qi Han, Yanqing Huang, Yanhui Wei, Jie Zi, Lidong Zhao, Zhimei Cai, Xuzhang Lu, Rong Xiao, Yanming Zhang, Xiaotian Yang, Hao Xu, Naitong Sun, Wanchuan Zhuang, Zhengdong Wu, Yuan Xia, Yanli Xu, Bin He, Wei Zhu, Fengling Min, Yongchun Chen, Banghe Ding, Peimin Shi, Jing Xie, Hua Tang, Zefa Liu, Bingzong Li, Yu Sun, Hongxia Qiu, Limin Duan, Elanora Dovat, Chunhua Song, Laszlo SzeKely, Sinisa Dovat, Zheng Ge

**Affiliations:** 1 Department of Hematology, Zhongda Hospital, School of Medicine, Southeast University, Institute of Hematology Southeast University, Nanjing, China; 2 Department of Hematology, The First People’s Hospital of Lianyungang, Lianyungang, China; 3 Department of Hematology, Changzhou No.2 People’s Hospital, Changzhou, China; 4 Department of Hematology, Huai’an Second People’s Hospital, Huai’an, China; 5 Department of Hematology, Yancheng No.1 People’s Hospital, Yancheng, China; 6 Department of Hematology, Yancheng Third People’s Hospital, Yancheng, China; 7 Department of Hematology, The Second People’s Hospital of Lianyungang, Lianyungang, China; 8 Department of Hematology, Jiangsu Taizhou People’s Hospital, Taizhou, China; 9 Department of Hematology, Nanjing First Hospital, Nanjing Medical University, Nanjing, China; 10 Department of Hematology, Northern Jiangsu People’s Hospital, Yangzhou, China; 11 Department of Hematology, Xuzhou No. 1 People’s Hospital, Xuzhou, China; 12 Department of Hematology, Affiliated Hospital of Yangzhou University, Yangzhou, China; 13 Department of Hematology, Huai’an First People’s Hospital, Huai’an, China; 14 Department of Hematology, Taixing People’s Hospital, Taizhou, China; 15 Department of Hematology, Xinghua City People’s Hospital, Taizhou, China; 16 Department of Hematology, The Second Affiliated Hospital of Soochow University, Suzhou, China; 17 Department of Geriatric Hematology, The First Affiliated Hospital of Nanjing Medical University, Nanjing, China; 18 Hershey Medical Center, Pennsylvania State University Medical College, Hershey, PA USA; 19 Division of Hematology, The Ohio State University Wexner Medical Center, the James Cancer Hospital, Columbus, OH USA; 20 Department of Clinical Pathology and Cancer Diagnostics, Karolinska University Hospital, Department of Laboratory Medicine, Division of Pathology, Karolinska Institute, Stockholm, Sweden

**Keywords:** Phase II trials, Chemotherapy

Acute myeloid leukemia (AML) is a heterogeneous hematologic malignancy with high mortality and poor outcome, especially for elderly/unfit (age ≥ 60 years or unfit patients who are ineligible to receive intensive chemotherapy) with adverse genetic and molecular abnormalities in the newly diagnosed (ND) and refractory/relapsed (R/R) patients.

Azacitidine (Aza), a hypomethylating agent, targets epigenetic gene silencing by inhibiting gene expression against malignant phenotypes. The complete remission (CR) rates of Aza combined regimens varied with different drugs, such as, Aza+Midostaurin (20.8%), Aza+Durvalumab (31.3%), Aza+Pracinostat (46%), Aza+Venetoclax (66.4%) in ND AML and Aza+Midostaurin (21.4%), Aza+Nivolumab (22%), Aza+Venetoclax (37.1%) in R/R AML [1–7].

Homoharringtonine (HHT) is a natural plant alkaloid isolated from *Cephalotaxus* and exhibits an anti-leukemic effect [8, 9]. Several studies revealed that HHT- combined regimens are effective and safe in patients with chronic myeloid leukemia of ND or failed prior therapy (imatinib, IFNa) [10–13]. It is reported that HHT in combination with cytarabine, aclarubicin/daunorubicin (HAA/HDA) achieved a higher CR than the standard “3 + 7” regimen (DA) in treating de novo AML [8]. HAG (HHT, low-dose cytarabine, G-CSF), which is called a priming regimen, was preferred to treat the elderly/unfit AML due to its low cardiac toxicity and well-tolerance. However, so far, it has not been well determined the efficacy and safety of adding Aza to the HAG regimen.

This is a multicenter, single-arm, phase 2 clinical trial done in 17 institutions in China. The trial was registered on ClinicalTrials.gov (NCT04248595). This study was approved by IEC for Clinical Research of Zhongda Hospital, Affiliated to Southeast University (2019ZDSYLL211-P01) and all patients provided written informed consent. Inclusion criteria were: 1) confirmed diagnosis of AML by the WHO criteria. 2) age ≥ 60 years or unfit patients who are ineligible to receive intensive chemotherapy who had at least one of the following coexisting conditions: [1] a history of congestive heart failure with an ejection fraction of 50% or less, or chronic stable angina [2] chronic respiratory disease with forced expiratory volume less than 65% in 1 s [3], Eastern Cooperative Oncology Group (ECOG) score of 2 or 3. 3) aspartate and alanine aminotransferase concentrations < 2 times the upper limit of normal, serum bilirubin concentration ≤ 35 μmol/L. 4) able to understand and provide written consent. Exclusion criteria were: 1) confirmed diagnosis of acute promyelocytic leukemia. 2) < 18 years. 3) co-existence with other non-hematological neoplasms. 4) infection of HIV. 5) lactating or pregnant women. 6) other medical or psychological diseases which investigators believed were not suitable to enter the trial.

A total of 112 patients [median age 65 (24–86) years)] were enrolled between Jan 2020 and Dec 2021, including 72 ND and 40 R/R (Fig. S1A). The 72 ND AML comprise 56 de novo AML [no history of myelodysplastic syndrome (MDS), myeloproliferative neoplasms (MPN), or exposure to potentially leukemogenic agents] and 16 secondary AML (s-AML, arising from preexisting myeloid neoplasms). Among them, 33 patients aged < 60 years were recruited according to the unfit criteria (Table S1). The risk categories of the 72 ND AML patients were classified into favorable-risk (19/72), intermediate-risk (31/72), and poor-risk (22/72).

Induction therapy consisted of Aza (75 mg/m^2^/d on days 1–7 subcutaneous) was given in combination with the HAG regimen [HHT 1 mg/m²/d on days 4–17 intravenous over 3 h, cytarabine 10 mg/m² every 12 h on days 4–17 subcutaneous, and G-CSF 200 μg/m²/d subcutaneous from day 4 until WBC > 10 × 10^9^/L(14-day HAG schedule) (38/112 patients); HHT 1 mg/m²/d on days 4–10 intravenous over 3 h, cytarabine 10 mg/m² every 12 h on days 4–10 subcutaneous, and G-CSF 200 μg/m²/d subcutaneous from day 4 until WBC > 10 × 10^9^/L(7-day HAG schedule) (74/112 patients)]. Patients who did not achieve CR/CRi (CR with incomplete blood count recovery) following the first cycle could receive a second cycle at the same doses and schedule. The patients who did not reach CR/CRi after the second cycle were withdrawn from the study. Post-remission therapy for enrolled patients, the Aza+HAG regimen was further given 4–6 cycles or until the disease progresses, two of whom underwent allo-SCT (one was 55 years with sAML from MDS, the other was 41 years with relapsed AML).

The detailed procedures, clinical endpoint, and assessments are in online supplemental methods. The gene mutations were screened in 103 patients using the targeted leukemia exome-seq panel including 58 genes. The detailed panel design, screening, data analysis; association of gene mutations with clinical responses, relapse status, risk groups, and other statistics are described in the online supplemental methods.

Of 112 patients, 73 (65.2%) achieved CR/CRi within the median time of 33.5d. Notably, 90.4% (66/73) of CR/CRi was obtained after the 1st cycle of Aza+HAG induction therapy. No statistical difference in CR/CRi rate between Aza+HAG (7-day) (62.2%, 46/74) vs Aza+HAG (14-day) (71.1%, 27/38) (*P* = 0.35). A higher CR/CRi rate of 79.2% in ND AML compared with 40.0% in R/R AML; and sAML (75.0%) obtained a similar high CR rate as de novo AML (80.4%) (Fig. 1A). In patients with favorable, intermediate or poor risk, the CR/CRi rate were 94.7%, 83.9%, and 59.1%, respectively (Fig. 1B). These data indicated that Aza+HAG regimen has high efficacy not only for ND de novo AML with favorable-intermediate risk, but also for elderly/unfit patients with sAML, poor-risk, and R/R AML.

**Fig. 1:**
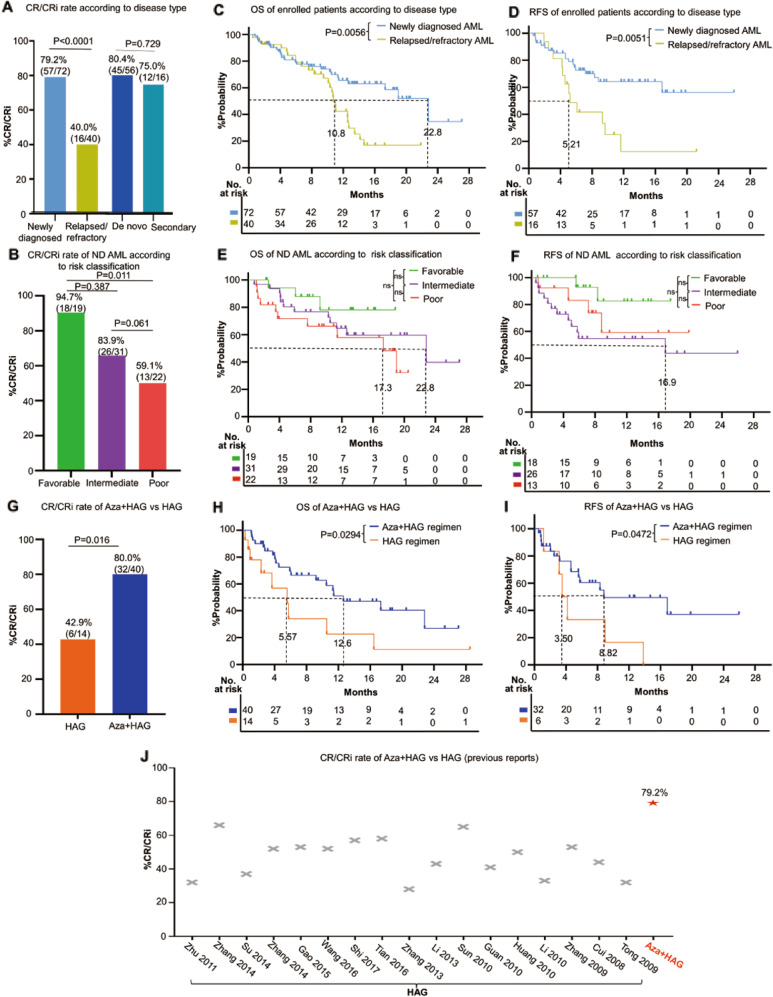
Clinical efficacy of Aza + HAG regimen. **A** CR/CRi rate of Aza+HAG regimen according to different disease types (newly diagnosed AML, relapsed/refractory AML, de novo AML, and secondary AML). **B** CR/CRi rate of Aza+HAG regimen of newly diagnosed AML patients according to different risk groups (favorable, intermediate, poor risk [ELN2017]). **C**, **D** OS (**C**) and RFS (**D**) curves of Aza+HAG regimen according to disease type. **E**, **F** OS (**E**) and RFS (**F**) curves of Aza+HAG regimen of newly diagnosed AML patients according to different risk groups. (**G**) CR/CRi rate of enrolled patients receiving Aza+HAG regimen versus HAG control. Patients from both the Aza+HAG and HAG control were derived from the same clinical institute (Zhongda Hospital, Nanjing, China), (**H**, **I**) OS (**H**) and RFS (**I**) curves of patients receiving the Aza+HAG regimen versus HAG control, (J) CR/CRi rate of Aza+HAG regimen were compared with previously reported CR/CRi rate of HAG regimen (data were derived from 17 studies, including 453 patients).

The median overall survival (OS) and relapse-free survival (RFS) in ND AML were 22.8 m and not reached, respectively, which were longer than R/R AML (median OS 10.8 m, *P* = 0.0056; median RFS 5.21 m, *P* = 0.0051) (Fig. 1C, D). De novo AML had a superior median OS (22.8 m) and RFS (not reached) compared with sAML (median OS 11.3 m, *P* = 0.01; median RFS 5.61 m, *P* = 0.0048) (Fig. S1B, C**)**. In patients with favorable, intermediate, or poor risk, median OS was not reached, 22.8 m, and 17.3 m (Fig. 1E); median RFS was not reached, 16.9 m, and not reached (Fig. 1F), respectively. These data suggested that better survival could be achieved with the Aza+HAG regimen, especially for elderly/unfit patients with ND de novo AML.

We further compared the efficacy of the Aza+HAG vs HAG regimen in our center (Zhongda Hospital). The baseline demographic and characteristics were generally balanced between the two cohorts (Table S2). The CR/CRi rate with the Aza+HAG regimen (80.0%) is significantly higher than that of the HAG control (42.9%) (*P* = 0.016) (Fig. 1G). The median OS and RFS with the Aza+HAG regimen were significantly longer than HAG control (12.6 m vs 5.57 m, *P* = 0.0294; 8.82 m vs 3.50 m, *P* = 0.0472), respectively (Fig. 1H, I). We also performed a meta-analysis including 453 elderly/unfit AML patients from 17 previous reported HAG studies and the estimated CR/CRi rate was 47.0% (Fig. 1J and S2). These data indicated that adding Aza to the HAG increases the clinical responses and outcomes for elderly/unfit AML.

The most common non-hematological adverse event (AE) was infection (58.0%). Common non-hematological AEs of grade 3 or higher includes: infection (33.9%), hemorrhage (9.82%), fatigue (5.36%), hypokalemia (3.57%), cardiac arrhythmia (1.79%), and fever (1.79%). For the hematological AE, the median duration of neutropenia and thrombocytopenia were 11 d (IQR, 7–19d) and 16d (IQR, 11–25d), 10 d (IQR, 6.25–18d), and 16 d (IQR, 10.25–24.75d), 12d (IQR, 8–21d) and 17d (IQR, 12–27d) for total, 7-day, and 14-day schedule, respectively. No differences were observed between the 7-day and 14-day schedule. The early deaths within 4 weeks of the induction treatment occurred in 1.79%. No patients discontinued the induction therapy due to hematological or non-hematological toxicities (Table S3). These data indicated that HHT had much lower cardiac toxicity and hematological suppression compared with daunorubicin in the standard regimen.

We detected 228 mutants involving 33 genes in 103 patients. The median mutation number per patient was 2.21 (range, 0–7) and 87.4% (90/103) of patients had more than one gene mutation. The most frequently mutated genes were *DNMT3A, TET2, and NPM1* (Fig. 2A). We observed the high CR/CRi rate in ND patients with mutated *BCOR* (100%,7/7), *KIT* (100%,3/3), *IDH1* (100%,7/7)*, NPM1* (93.3%,14/15), *ASXL1* (88.9%,8/9)*, DNMT3A* (80. 0%,16/20), *RUNX1* (75.0%,6/8), *FLT3* (71.4%,5/7) and *TET2* (71.4%,10/14) (Fig. 2B) (Table S4). We also found the variant allele fraction (VAF) of mutants was dramatically reduced (<0.01%), particularly with mutations of *FLT3*, *IDH1*, *ASXL1*, and *NPM1* (Fig. 2C-F). OS and RFS in patients with *FLT3* or *ASXL1* mutation showed no inferior tendency, compared with those without *FLT3* or *ASXL1* mutation (Fig. S3A-D); whereas patients with *IDH1* mutation showed a better tendency in RFS, compared with patients with *IDH1* wild type (Fig. S3E, F). These data suggested that this scheme could overcome the poor prognosis associated with unfavorable molecular abnormalities.

**Fig. 2:**
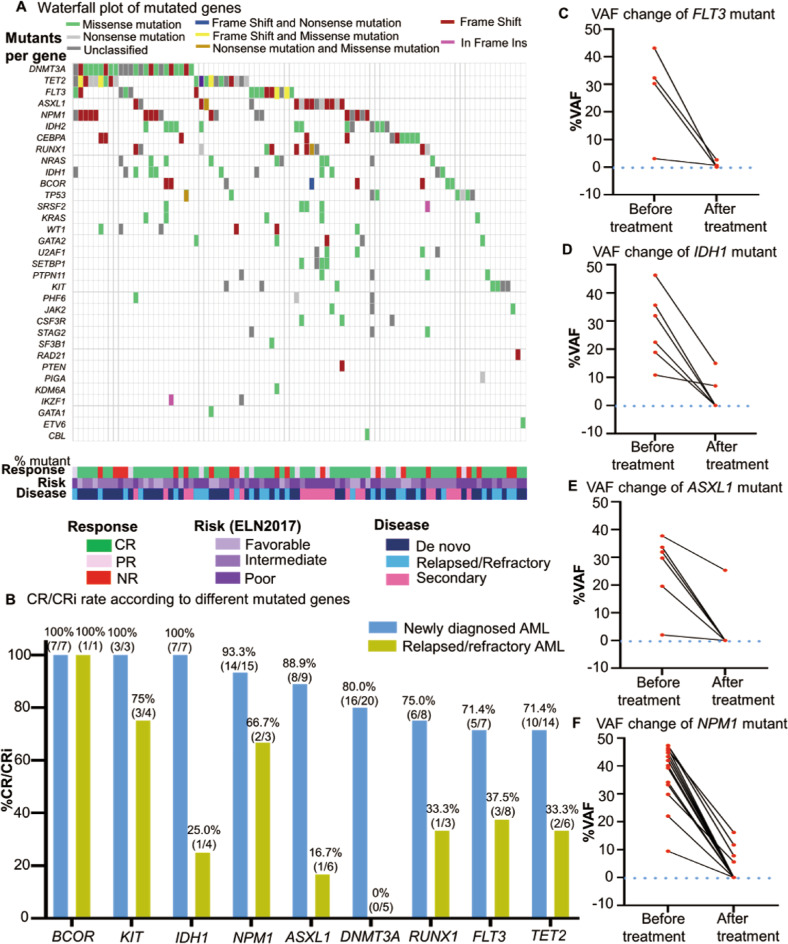
Gene mutation profiling and effect on the clinical efficacy of Aza + HAG regimen. **A** Landscape of mutations detected in 90 of 112 enrolled patients. Each row represents a gene, and each column corresponds to a participant. The sidebar plots the number of mutations detected for each gene. **B** CR/CRi rate in patients harbored with different gene mutants according to different disease types. **C** Clearance of *FLT3* mutant (TKD or ITD) was represented by dynamic changes in VAF before and after treatment in 4 CR/CRi patients who harbored *FLT3* mutant. **D** Clearance of *IDH1* mutant was represented by dynamic changes in VAF before and after treatment in 6 CR/CRi patients who harbored *IDH1* mutant. **E**) Clearance of *ASXL1* mutant was represented by dynamic changes in VAF before and after treatment in 6 CR/CRi patients who harbored *ASXL1* mutant. **F** Clearance of *NPM1* mutant was represented by dynamic changes in VAF before and after treatment in 14 CR/CRi patients who harbored *NPM1* mutant.

Recent studies reported the CR rates of patients treated with the Aza plus *IDH1* inhibitor Ivosidenib (60.9%) [14] in *IDH1*-mutant ND AML or HMA + Venetoclax (71%) in *IDH1/2-*mutant AML [15]. This trial showed a high CR/CRi rate of 100% in the *IDH1*-mutant AML and the *IDH1* mutant was dramatically reduced after the first cycle of Aza+HAG, suggesting that the patients with the *IDH1* mutation could achieve a remarkable deep and durable remission upon Aza+HAG treatment.

In summary, this trial demonstrated that the Aza+HAG regimen is a cost-effective first-line therapy with high efficacy and well tolerance for elderly/unfit AML. This trial provides a rationale for further expanding the patients for randomized clinical controlled studies and guiding suggestions for the clinical use of this novel combination therapy.

## Supplementary information

Supplemental methods_tables_figures

## Data Availability

All data generated or analyzed during this study are included in this published article.
