# Supplementary material for: High efficacy of Azacitidine plus HAG in acute myeloid leukemia: an open-label, single-arm, multi-center, phase 2 study
Source: Blood Cancer J. 2022 Oct 28;12(10):145. doi: 10.1038/s41408-022-00740-3 (PMC9616883; doi:10.1038/s41408-022-00740-3)
Supplement: Supplementary file 1 — Supplemental methods_tables_figures [file 41408_2022_740_MOESM1_ESM.pdf]

Supplemental Material
High efficacy of Azacitidine plus HAG in acute myeloid leukemia: an open-label, single-arm, multi-center, phase 2 study

Jun Li[1]*, Qi Han[1]*, Yanqing Huang[1]*, Yanhui Wei[1], Jie Zi[1], Lidong Zhao[2], Zhimei Cai[2], Xuzhang Lu[3], Rong Xiao[3], Yanming Zhang[4], Xiaotian Yang[4], Hao Xu[5], Naitong Sun[6], Wanchuan Zhuang[7], Zhengdong Wu[8], Yuan Xia[8], Yanli Xu[9], Bin He[10], Wei Zhu[11], Fengling Min[12], Yongchun Chen[12], Banghe Ding[13], Peimin Shi[14], Jing Xie[14], Hua Tang[15], Zefa Liu[15], Bingzong Li[16], Yu Sun[16], Hongxia Qiu[17], Limin Duan[17], Elanora Dovat[18], Chunhua Song[18, 19#], Laszlo SzeKely[20], Sinisa Dovat[18], Zheng Ge[1#]

[1]Department of Hematology, Zhongda Hospital, School of Medicine, Southeast University, Institute of Hematology Southeast University, Nanjing, China. [2]Department of Hematology, The First People's Hospital of Lianyungang, Lianyungang, China. [3]Department of Hematology, Changzhou No.2 People's Hospital, Changzhou, China. [4]Department of Hematology, Huai'an Second People's Hospital, Huai'an, China. [5]Department of Hematology, Yancheng No.1 People's Hospital, Yancheng, China. [6]Department of Hematology, Yancheng Third People's Hospital, Yancheng, China. [7]Department of Hematology, The Second People's Hospital of Lianyungang, Lianyungang, China. [8]Department of Hematology, Jiangsu Taizhou People's Hospital, Taizhou, China. [9]Department of Hematology, Nanjing First Hospital, Nanjing Medical University, Nanjing, China. [10]Department of Hematology, Northern Jiangsu People's Hospital, Yangzhou, China. [11]Department of Hematology, Xuzhou No. 1 People's Hospital, Xuzhou, China. [12]Department of Hematology, Affiliated Hospital of Yangzhou University, Yangzhou, China. [13]Department of Hematology, Huai'an First People's Hospital, Huai'an, China. [14]Department of Hematology, Taixing People's Hospital, Taizhou, China. [15]Department of Hematology, Xinghua City People's Hospital, Taizhou, China. [16]Department of Hematology, The Second Affiliated Hospital of Soochow University, Suzhou, China. [17]Department of Geriatric Hematology, The First Affiliated Hospital of Nanjing Medical University, Nanjing, China. [18]Hershey Medical Center, Pennsylvania State University Medical College, Hershey, USA. [19]Division of Hematology, The Ohio State University Wexner Medical Center, the James Cancer Hospital, Columbus, USA. [20]Department of Clinical Pathology and Cancer Diagnostics, Karolinska University Hospital, Department of Laboratory Medicine, Division of Pathology, Karolinska Institute, Sweden.

Running title: High efficacy of Aza+HAG regimen in AML

*These authors contributed equally to the work.

[#]Co-correspondence to:
Zheng Ge, M.D., Ph.D.
Department of Hematology
Zhongda Hospital, School of Medicine, Southeast University
Institute of Hematology Southeast University
87 Dingjiaqiao Street, Nanjing 210009, China,
Telephone: +86 25-83262468

Fax: +86 25-83262471
E-mail: zhengge@seu.edu.cn
ORCID: orcid. org/0000-0001-8028-1612

Chunhua Song, M.D., Ph.D.
Hershey Medical Center,
Pennsylvania State University Medical College
The James Comprehensive Cancer Center
Ohio State University, Division of Hematology
536 Biomedical Research Tower, 460 W. 12th Ave.
Columbus, OH43210, USA
Telephone: 614-2928715, FAX: 614-293-7526
E-mail: chunhua.song@oscmc.edu
ORCID: 0000-0002-4081-2543

Supplemental Methods

Clinical endpoint and assessments

The primary endpoint was the composite complete remission (complete remission [CR] or complete remission with incomplete hematologic recovery [CRi]). Secondary endpoints were overall survival (OS) defined as the time from study entry to death from any cause), relapse-free survival (RFS) is defined as the time from achieving a CR until disease recurrence or death), adverse events (AE) (including hematological and non-hematological AE defined as any unfavorable and unintended signs including an abnormal laboratory finding, symptom, or disease). Treatment failure was defined as not achieving CR or CRi after two cycles of induction therapy.

Prespecified correlative assessments included targeted gene panel sequencing to assess associations between somatic mutation patterns and therapeutic responses as well as disease progression.

Response to therapy was monitored by analysis of blood and bone marrow aspirates. Response assessment was done at the end of cycles 1 and 2 (no CR/CRi after cycle 1), and then after every 2 cycles of consolidation and every 3 months during maintenance to confirm ongoing response. Responses were categorized based on the revised International Working Group criteria for AML[1, 2].

Minimal residual disease (MRD) assessment by multicolor flow cytometry (MFC) was done on pretreatment bone marrow and all subsequent bone marrow examinations with the assay sensitivity of 0.01% as previously described[3]. Bone marrow samples were obtained at diagnosis and evaluated for the presence of cytogenetic and molecular aberrations. For documentation of mutations, the entire coding sequences of 58 genes known to be frequently mutated in myeloid malignancies were sequenced with a targeted leukemia exome-seq panel (**Table S5)** as described below and supplemental methods.

AE and laboratory values, graded according to the Common Terminology Criteria for Adverse Events version 4.0, were evaluated at least once every cycle during induction and consolidation and then at least every 3 months during the maintenance.

Sample size estimation

In this trial, the optimal Simon's two-stage design method was used to determine the sample size. A composite complete remission rate ≤ 40% (p0) would be considered a null hypothesis (the outcome was unacceptable). While a composite complete remission rate of higher than 60% (p1) would grant the regimen for further exploration. An estimation power of 95%, with a significance level of 2.5% was used to test the hypothesis.

Accordingly, 35 participants in the 1st stage with additional 54 participants in the 2nd stage where needed. Aza+HAG regimen would be discontinued if 15 or fewer achieved CR/CRi in the 1st stage. Patients' enrollment in the 2nd stage would be held until the outcome of the 1st stage interim analysis. If the Aza+HAG regimen continued, the activity of treatment would be considered a null hypothesis if less than 44 out of 90 enrolled cases achieved CR/CRi. Considering a 20% dropout rate, the estimated sample size was 112 patients.

HAG regimen control

A total of 14 ND AML patients with HAG induction regimen (homoharringtonine, cytarabine, G-CSF) from Jan 2016 to Aug 2019 in Zhongda Hospital (Nanjing, China) were enrolled as the control. The 14 patients include 12 de novo and 2 secondary AML.

HAG regimen consisted of HHT 1mg/m^2/d on days 1-14 intravenous over 3 hours, cytarabine 10mg/m^2 every 12 hours on days 1-14 subcutaneous, and G-CSF 200μg/m^2/d subcutaneous from day 1 until WBC>10×10^9/L(14-day HAG schedule) (4/14 patients); HHT 1mg/m^2/d on days 1-7 intravenous over 3 hours, cytarabine 10mg/m^2 every 12 hours on days 1-7 subcutaneous, and G-CSF 200μg/m^2/d subcutaneous from day 1 until WBC>10×10^9/L(7-day HAG schedule) (10/14 patients).

Baseline demographic and disease characteristics were generally balanced between 40 ND AML patients with Aza+ HAG regimen versus 14 ND AML patients with HAG regimen in the same center (Zhongda Hospital) (**Table S2**).

Comparison of CR/CRi rate between Aza+HAG and HAG group was performed using the

Chi-square test or Fisher exact test (when the sample size is small and with less degree of freedom). The comparison of OS and RFS between the two groups was performed using Kaplan-Meier estimates with the log-rank test. A threshold P-value < 0.05 was considered a statistically significant difference.

Targeted exome-seq panel for gene mutation screening in AML patients

A leukemia targeted-exome-seq panel including 58 genes was used for screening the gene mutations in 103 enrolled patients by next-generation sequencing (NGS) before and after the induction therapy[4, 5]. The target genes in the panel are listed in **Table S5**.

Agilent SureSelect Human All Exon V4+UTRs (Agilent) was used for the coding exons plus UTRs of target genes. Probes for each exon of each target gene are designed on NCBI (https://www.ncbi.nlm.nih.gov/). The targeted exome-seq method is performed as reported[6]. Briefly, the genomic DNA was isolated from bone marrow samples with the genomic DNA isolation kit (Qiagen, Hilden, Germany). All DNA samples were sheared with a Covaris E220 instrument generating approximately 260 bp DNA fragments. The fragmented DNA was processed into Illumina-compatible sequencing libraries using Kapa Hyper Prep Kit (Illumina, San Diego, CA, USA). Each library was uniquely barcoded and captured by the leukemia panel probes, followed by PCR amplification and sequencing on a HiSeq 2500 (Illumina) with 2x100 bp reads. The sequencing reads were aligned to the human genome by following Broad Institute's GATK best-practice pipeline to call germline short variants (SNPs and Indels). Called variants were annotated using ANNOVAR (version 2.3). Exonic variants with exonic, nonsynonymous, stop-gain, or stop-loss, novel SNPs, and with predicted deleterious/damaging functions were manually surveyed by IGV to confirm.

The association of gene mutations with clinical response, relapse, and risk status was analyzed with R 4.0.1 software and depicted as a waterfall figure. The association of the gene mutations with OS and RFS was also evaluated by the Kaplan-Meier method[7-9].

Meta-analysis of HAG regimen in treating elderly AML patients

To evaluate the clinical response of the HAG regimen in treating an elderly patient with AML,

we conducted a meta-analysis of the HAG regimen by carefully screening MEDLINE, PubMed, EMBASE, and CNKI (Chinese) databases. Inclusion criteria were: 1) included unfit AML (previously untreated) patients (age over 60 years or ineligible for receiving standard chemotherapy) who received HAG regimen; 2) reported the clinical responses (CR or CRi).

A total of 453 patients from 17 studies [10-26] were finally included in this study, meta-analysis was conducted on the R 4.0.1 platform (meta-package). Funnel plot was routinely used to detect publication bias. To make the included data normalized, we used the Sharpiro- Wilk normality test to choose the best transformation method (arcsine conversion, free-man tukey conversion, logistic conversion, logarithmic conversion). The fixed-effect model will be applied if the heterogeneity is less than 25% (I^2). Otherwise, a random-effect model will be applied.

Statistical analysis

The distribution of survival was estimated with the use of the Kaplan-Meier method. The lower limit and upper limit of 95% confidence interval were calculated by the Wilson method. The student t-test was used to identify differences between groups. Categorical parameters were compared with the chi-squared test or Fisher's exact test. Statistical analysis was performed on STATA 16.0 software.

Data sharing statement

The patient datasets for the current study are not publicly accessible following local health research ethics protocols; however, they may be available from the corresponding author.

De-identified individual-level data and the data dictionary will be made available to qualified researchers who present study protocols, which will require approval by the institute health research ethics committee and principal investigator. These data will only be made available from study sites at which the institution and ethics review board allow such release.

Reference

1. Creutzig U, Kaspers GJ. Revised recommendations of the International Working Group for diagnosis, standardization of response criteria, treatment outcomes, and reporting standards for therapeutic trials in acute myeloid leukemia. J Clin Oncol. 2004;22(16):3432-3.
2. Cheson BD, Bennett JM, Kopecky KJ, Buchner T, Willman CL, Estey EH, et al. Revised recommendations of the International Working Group for Diagnosis, Standardization of

Response Criteria, Treatment Outcomes, and Reporting Standards for Therapeutic Trials in Acute Myeloid Leukemia. J Clin Oncol. 2003;21(24):4642-9.
3. Ravandi F, Jorgensen JL, O'Brien SM, Jabbour E, Thomas DA, Borthakur G, et al. Minimal residual disease assessed by multi-parameter flow cytometry is highly prognostic in adult patients with acute lymphoblastic leukaemia. Br J Haematol. 2016;172(3):392-400.
4. Song C, Pan X, Ge Z, Gowda C, Ding Y, Li H, et al. Epigenetic regulation of gene expression by Ikaros, HDAC1 and Casein Kinase II in leukemia. Leukemia. 2016;30(6):1436-40.
5. Song C, Gowda C, Pan X, Ding Y, Tong Y, Tan BH, et al. Targeting casein kinase II restores Ikaros tumor suppressor activity and demonstrates therapeutic efficacy in high-risk leukemia. Blood. 2015;126(15):1813-22.
6. Clark MJ, Chen R, Lam HY, Karczewski KJ, Chen R, Euskirchen G, et al. Performance comparison of exome DNA sequencing technologies. Nat Biotechnol. 2011;29(10):908-14.
7. Ge Z, Gu Y, Han Q, Sloane J, Ge Q, Gao G, et al. Plant homeodomain finger protein 2 as a novel IKAROS target in acute lymphoblastic leukemia. Epigenomics. 2018;10(1):59-69.
8. Ge Z, Gu Y, Han Q, Zhao G, Li M, Li J, et al. Targeting High Dynamin-2 (DNM2) Expression by Restoring Ikaros Function in Acute Lymphoblastic Leukemia. Sci Rep. 2016;6:38004.
9. Guo X, Zhang R, Liu J, Li M, Song C, Dovat S, et al. Characterization of LEF1 High Expression and Novel Mutations in Adult Acute Lymphoblastic Leukemia. PLoS One. 2015;10(5):e0125429.
10. Zhu JH. Therapeutic effect of HAG regimen combined with traditional Chinese medicine on senile acute myeloid leukemia. Mod J Integr Tradit Chin Med Wes Med. 2011;20(36):4637-8. (Article in Chinese)
11. Zhang P. Observation and nursing care of elderly patients with acute myeloid leukemia treated with pre-excitation scheme. Chin J Pharm Econ. 2014;9(06):141-142. (Article in Chinese)
12. Su J. Comparison of therapeutic effects of CAG and HAG regimen on elderly patients with acute myeloid leukemia. Chin Pharm. 2014;23(15):114-116. (Article in Chinese)
13. Zhang YH. Clinical observation of HAG regimen in the treatment of newly diagnosed elderly acute myeloid leukemia. Chin J Cont Ende Dise. 2014;29(S2):185-186. (Article in Chinese)
14. Gao XY, Liu M, Tian XQ, Lv RL. Therapeutic effect of HAG regimen on senile acute myeloid leukemia. Shaanxi Med J, 2015;44(04):444-446. (Article in Chinese)
15. Wang YM. Therapeutic effect of HAG regimen on elderly patients with acute myeloid leukemia. Zhongguo Nongcun Weisheng. 2016;(20):92-94. (Article in Chinese)
16． Shi HY, Liu ZG, Li J, Hu R, Yang Y, Wang HT. Comparison of therapeutic effects of HAG and HA regimen in the treatment of newly diagnosed elderly M2 acute myeloid leukemia. J Lab Med Clin. 2017; 14(03):406-408. (Article in Chinese)
17. Tian PJ, Zhu WM, Wang XJ, Chen F, Chen SJ. Clinical study of HAG preexcitation regimen in the treatment of 33 elderly patients with hypoproliferative acute myeloid leukemia. Pract Geriatr. 2016;30(04):348-349. (Article in Chinese)
18. Zhang YH, Meng J, He EX, Sun C, Li YF, Wang YY, et al. Clinical efficacy of homoharringtonine + cytarabine + granulocyte colony stimulating factor regimen combined

with elemene emulsion in the treatment of newly diagnosed elderly acute myeloid leukemia. Chin J Gerontol. 2013; 33(24):6138-6139. (Article in Chinese)
19. Li Q, Chen FH. Comparison of therapeutic effects of CAG and HAG regimen in the treatment of newly diagnosed elderly acute myeloid leukemia. Acta Acad Med Shantou Med. 2013;26(01):28-29. (Article in Chinese)
20. Sun RY. Clinical observation of HAG regimen in the treatment of senile acute myeloid leukemia. J Mod Oncol. 2010;18(10):2055-2056. (Article in Chinese)
21. Guan JM, Zhao WP, Xu H. Therapeutic effect of HAG regimen on senile acute myeloid leukemia. Chin J Pract Dign Treat. 2010;24(10):1015-1016. (Article in Chinese)
22. Huang K, Cao J, Gao F. Clinical analysis of HAG regimen in the treatment of senile acute myeloid leukemia. Contemp Med. 2010;16(31):105. (Article in Chinese)
23. Li JH. Therapeutic effect of HAG regimen on senile acute myeloid leukemia. Aerosol Med. 2010;21(09):1601. (Article in Chinese)
24. Zhang L, Su AL, Hu MQ, Zhang XQ, Zhang XZ, Xu YL. Clinical observation of individualized low-dose HAG regimen in the treatment of elderly acute myeloid leukemia. J Nanjing Med Univ. 2009; 29(12):1785-1786. (Article in Chinese)
25. Cui JY, Ran XH, Xia BS, Ren CA, Wang BH. Therapeutic effect of HAG regimen on senile acute myeloid leukemia. Chin Pract Med. 2008;(20):88-89. (Article in Chinese)
26. Tong JS, Yuan CJ. Clinical analysis of HAG regimen in the treatment of senile acute myeloid leukemia.Chin J Gerontol. 2009;29(03):362-363. (Article in Chinese)

Supplemental Tables

Table S1 Baseline characteristics of patients enrolled in Aza+ HAG regimen

	**Baseline characteristics**	
	**Characteristics(n=112)**	**Participants N (%); Median [IQR]**
**Sex**	Male	57(50.9)
	Female	55(49.1)
**Race or ethnicity**	Asian	112(100)
	White	0
	Black	0
	other	0
**Age**	Median	65[57.3-70.8]
	<60y	33(29.5)
	≥60y	79(70.5)
**Blood cell counting**	Median WBC (10^9/L)	5.2[2.20-21.8]
	Median Hemoglobin (g/L)	73[62.0-90.5]
	Median PLT (10^9/L)	50[24.5-96.8]
**FAB classification**	M0	1(0.893)
	M1	9(8.04)
	M2	61(54.5)
	M3	0
	M4	7(6.25)
	M5	27(24.1)
	M7	1(0.893)
	Unclassified	6(5.36)
**Diagnosis**	**Newly diagnosed**	72(64.3)
	De novo	56(50.0)
	Secondary	16(14.3)
	Favorable*	19(17.0)
	Intermediate*	31(27.7)
	Poor*	22(19.6)
	**Relapsed/refractory**	40(35.7)
**Mutation**	*DNMT3A*	25(24.3)
	IDH1/2	23(22.3)
	TET2	20(19.4)
	NPM1	18(17.5)
	FLT3	15(14.6)
	ASXL1	15(14.6)
	CEBPA	11(10.7)
	RUNX1	11(10.7)
	NRAS	10(9.71)
	TP53	9(8.74)
	BCOR	8(7.77)
	KIT	7(6.80)

Data are n (%), unless otherwise stated.

***Risk classification was evaluated by ELN2017 risk category.**

Abbreviations: FAB classification=French–American–British classification; WBC: white blood cell; PLT: platelet.

Table S2 The baseline characteristics between the patients treated with Aza+HAG and HAG regimen

		Aza+HAG	HAG	P
		40(100)	14(100)	
Mean age (SD; years)		65.8(10.8)	68.4(12.8)	0.4672
Disease type	De novo AML	28(70.0)	12(85.7)	0.311
	Secondary AML*	12(30.0)	2(14.3)	
Sex	Male	21(52.5)	6(42.9)	0.535
	Female	19(47.5)	8(57.1)	
Median WBC counting (10^9/L; IQR)		6.0 (1.95-19.8)	7.38 (2.49-15.6)	0.4937
Median Plt counting (10^9/L; IQR)		66.0 (37.3-103.5)	53 (18.5-124)	0.9403
Median Hemoglobin (g/L; IQR)		73.5 (62.0-91.8)	82 (72.8-97.8)	0.2032
ELN2017 risk category	Favorable	12(30.0)	1(7.14)	0.1077
	Intermediate	13(32.5)	4(28.6)	
	Poor	15(37.5)	8(57.1)	
	Unclassified	0	1(7.14)	
FAB classification	M0	0	1(7.14)	0.290
	M1	4(10)	0	
	M2	24(60)	10(71.4)	
	M3	0	0	
	M4	0	0	
	M5	11(27.5)	3(21.4)	
	M6	0	0	
	M7	1(2.5)	0	
	Not established	0	0	
Mutated genes	Mutation of *FLT3*	7(17.5)	2(14.3)	0.999
	Mutation of *NPM1*	14(35.0)	1(7.14)	0.10
	Mutation of *TP53*	6(15.0)	1(7.14)	0.662
	Mutation of *KIT*	3(7.50)	1(7.14)	0.999

Data are n (%), unless otherwise stated,

*secondary AML: AML arising from preexisting myeloid neoplasms, including myelodysplastic syndrome, myeloproliferative neoplasms, or exposure to potentially leukemogenic agents.

Table S3 Adverse events of Aza+ HAG regimen in enrolled patients

<table>
<tr><th colspan="2">AEs (adverse events)</th><th colspan="3">patients (n=112)</th></tr>
<tr><td rowspan="13">Non-Hematologic AEs</td><td></td><td>All grades</td><td colspan="2">Grades ≥3</td></tr>
<tr><td>Constipation</td><td>7(6.25)</td><td colspan="2">0</td></tr>
<tr><td>Diarrhea</td><td>6(5.36)</td><td colspan="2">1(0.893)</td></tr>
<tr><td>Vomiting</td><td>10(8.93)</td><td colspan="2">1(0.893)</td></tr>
<tr><td>Hypokalemia</td><td>10(8.93)</td><td colspan="2">4(3.57)</td></tr>
<tr><td>Peripheral edema</td><td>0</td><td colspan="2">0</td></tr>
<tr><td>Fatigue</td><td>23(20.5)</td><td colspan="2">6(5.36)</td></tr>
<tr><td>Hemorrhage</td><td>24(21.4)</td><td colspan="2">11(9.82)</td></tr>
<tr><td>Cardiac arrythmia</td><td>3(2.68)</td><td colspan="2">2(1.79)</td></tr>
<tr><td>Infection</td><td>65(58.0)</td><td colspan="2">38(33.9)</td></tr>
<tr><td>Nausea</td><td>21(18.8)</td><td colspan="2">0</td></tr>
<tr><td>Alanine/aspartate transaminase elevation</td><td>10(8.93)</td><td colspan="2">1(0.893)</td></tr>
<tr><td>Fever</td><td>38(33.9)</td><td colspan="2">2(1.79)</td></tr>
<tr><td>Early mortality</td><td>Died within 4 weeks</td><td colspan="3">2(1.79)</td></tr>
<tr><td rowspan="5">Hematologic AEs</td><td>Median duration of neutropenia (IQR; days)</td><td colspan="3">11(7-19)</td></tr>
<tr><td>Median duration of thrombocytopenia (IQR; days)</td><td colspan="3">16(11-25)</td></tr>
<tr><td></td><td>Aza + 7-day HAG</td><td>Aza + 14-day HAG</td><td>P</td></tr>
<tr><td>Median duration of neutropenia (IQR; days)</td><td>10(6.25-18)</td><td>12(8-21)</td><td>0.3141</td></tr>
<tr><td>Median duration of thrombocytopenia (IQR; days)</td><td>16(10.25-24.75)</td><td>17(12-27)</td><td>0.5617</td></tr>
</table>

Data are n (%), unless otherwise stated.

Table S4 Clinical responses in patients with different gene mutations

	**Total**					**Newly diagnosed**				
Gene	CR/CRi	PR	NR	Total	CR/CRi rate	CR/CRi	PR	NR	Total	CR/CRi rate
BCOR	8	0	0	8	100%	7	0	0	7	100%
NPM1	16	1	1	18	88.9%	14	0	1	15	93.3%
KIT	6	0	1	7	85.7%	3	0	0	3	100%
IDH1	8	1	2	11	72.7%	7	0	0	7	100%
CEBPA	8	1	2	11	72.7%	7	0	0	7	100%
DNMT3A	16	3	6	25	64.0%	16	1	3	20	80.0%
RUNX1	7	3	1	11	63.6%	6	1	1	8	75.0%
TET2	12	3	5	20	60.0%	10	1	3	14	71.4%
ASXL1	9	5	1	15	60.0%	8	1	0	9	88.9%
FLT3	8	3	4	15	53.3%	5	1	1	7	71.4%
IDH2	6	4	2	12	50.0%	6	2	1	9	66.7%
NRAS	4	2	4	10	40.0%	4	1	2	7	57.1%
TP53	1	3	5	9	11.1%	1	2	3	6	16.7%

Table S5 The Leukemia Panel for next generation sequencing

ABL1	BRAF	CEBPA	ETV6	HRAS
ANKRD26	CALR	CSF3R	EZH2	IDH1
ASXL1	CBL	CUX1	FLT3	IDH2
ATRX	CBLB	DDX41	GATA1	IKZF1
BCOR	CBLC	DNMT3A	GATA2	JAK2
BCORL1	CDKN2A	ETNK1	GNAS	JAK3
KDM6A	NPM1	PTEN	SMC1A	TP53
KIT	NRAS	PTPN11	SMC3	U2AF1
KMT2A	PDGFRA	RAD21	SRSF2	WT1
KRAS	PHF6	RUNX1	STAG1	ZRSR2
MPL	PIGA	SETBP1	STAG2	
NF1	PPM1D	SF3B1	TET2	

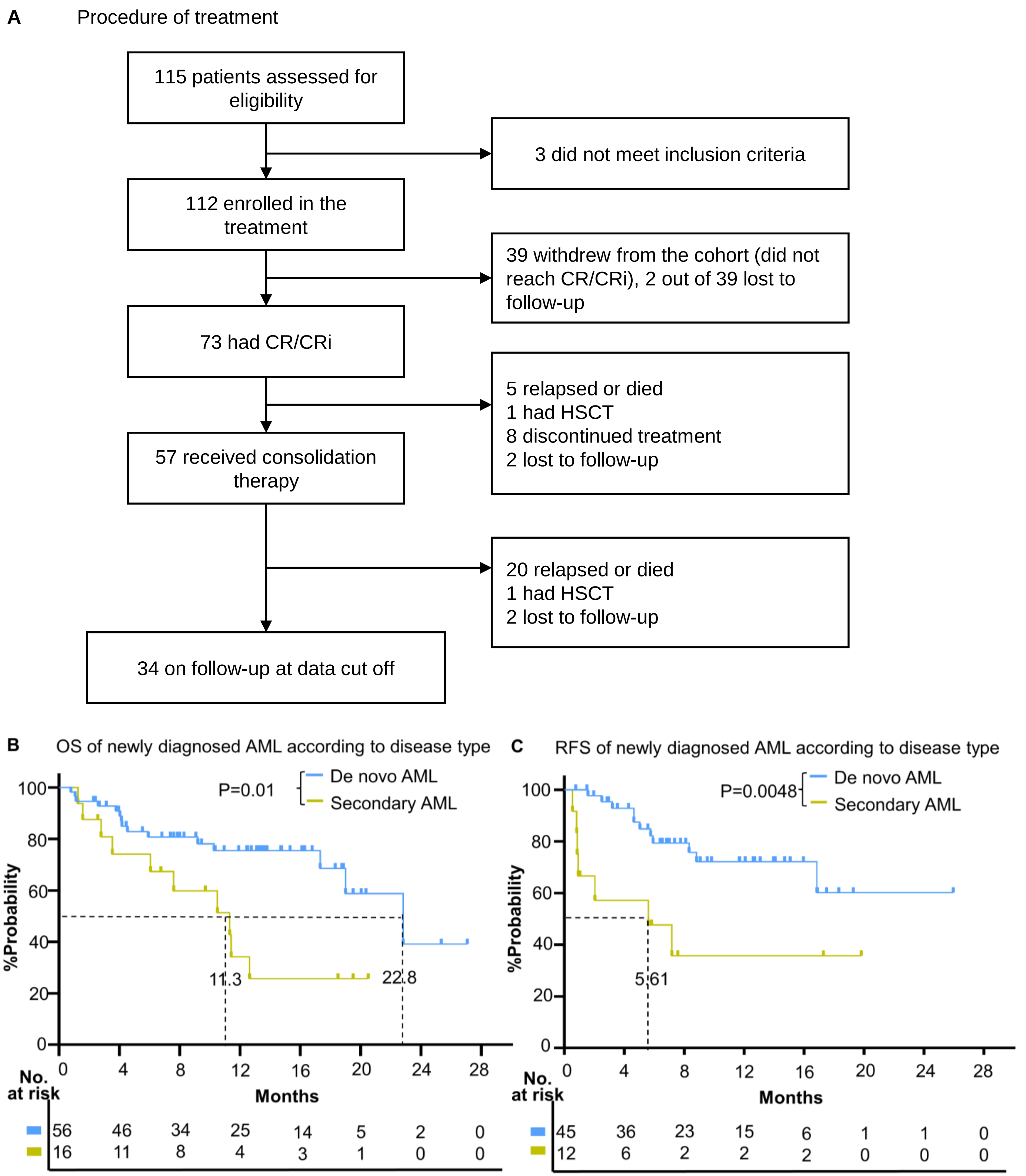

Fig S1 Clinical procedure of Aza +HAG regimen in this trial (A), a total of 115 AML patients were screened, 3 of them were not stratified according to the inclusion criteria. Finally, 112 patients were enrolled in this trial. Patients withdrawn from the cohort were followed for survival; OS (B) and RFS (C) curve of newly diagnosed AML patients versus secondary AML patients (arising from preexisting myeloid neoplasms).

A Meta-analysis result of HAG regimen in treating old/unfit newly diagnosed AML patients

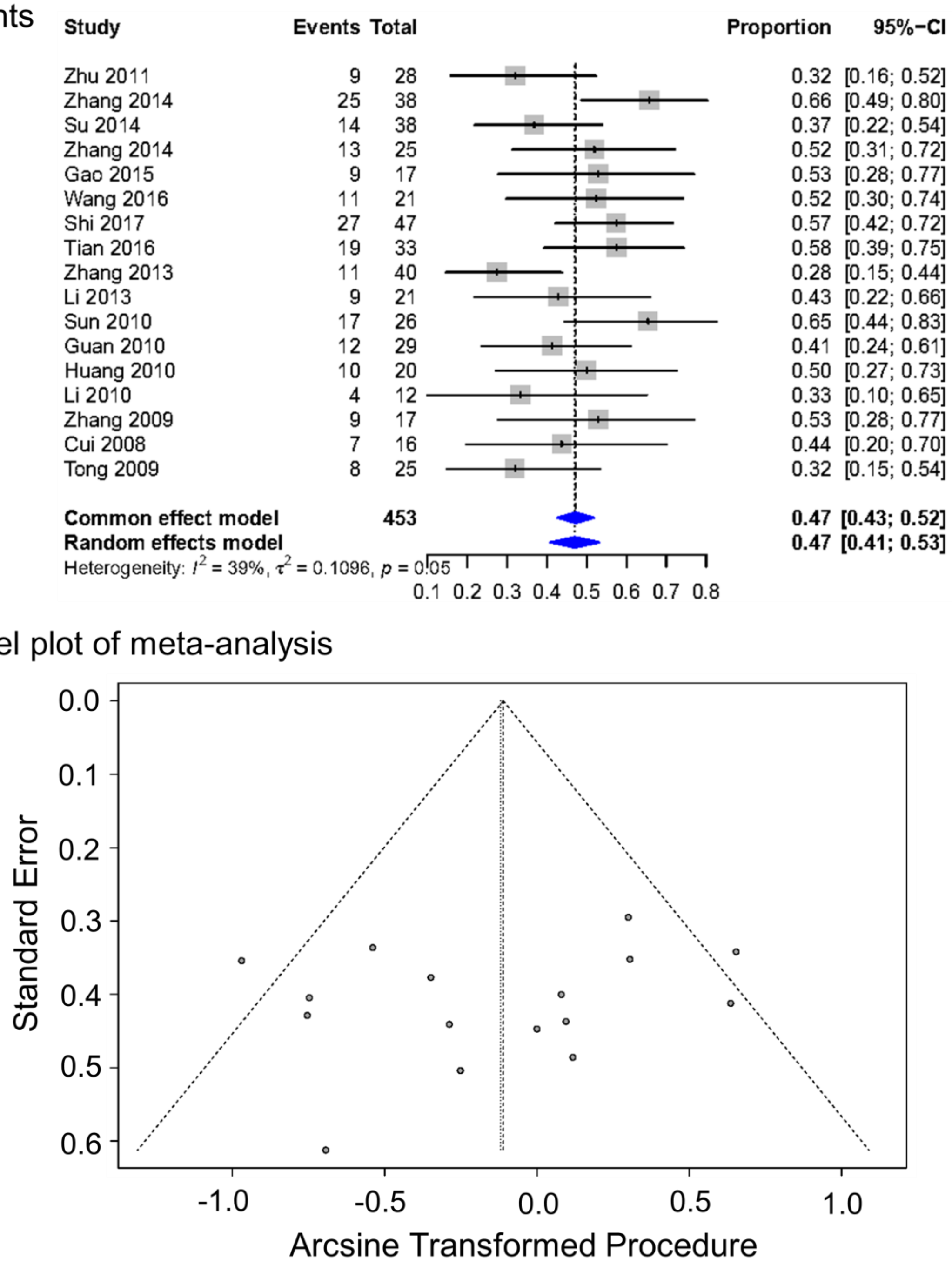

Fig S2 Meta-analysis result of HAG regimen (homoharringtonine, cytarabine, G-CSF) in treating old patients with newly diagnosed AML. (A) A total of 453 old/unfit AML patients (ineligible to receive intensive chemotherapy) from 17 studies were included, the CR/CRi rate of the HAG regimen was 47.0% (random-effects model, 95%CI, 41.0% to 53.0%) ; (B) Funnel plot of included 17 studies, no obvious bias was observed (linear regression test: t=-0.77, p=0.4559).

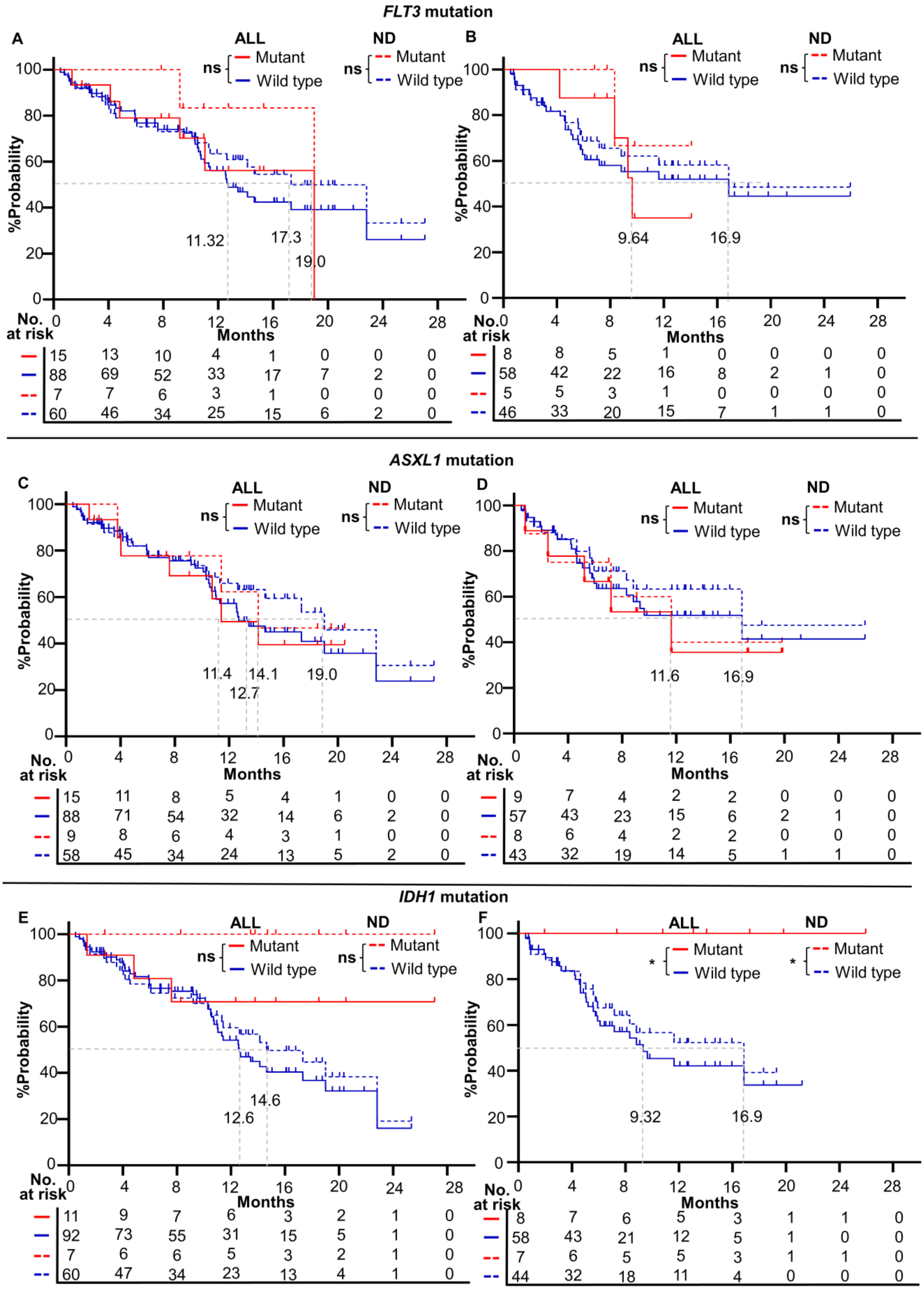

Fig S3 Survival of enrolled AML patients with the indicated gene mutants OS (A) and RFS (B) in patients with *FLT3* mutation (ITD or TKD) versus *FLT3* wild type. OS (C) and RFS (D) in patients with *ASXL1* mutation versus *ASXL1* wild type. OS (E) and RFS (F) in patients with *IDH1* mutation versus *IDH1* wild type. *P<0.05.
